# The Health Effects of Dietary Nitrate on Sarcopenia Development: Prospective Evidence from the UK Biobank

**DOI:** 10.3390/foods14010043

**Published:** 2024-12-27

**Authors:** Jigen Na, Yuefeng Tan, Yanan Zhang, Xiaona Na, Xiaojin Shi, Celi Yang, Zhihui Li, John S. Ji, Ai Zhao

**Affiliations:** 1 Vanke School of Public Health, Tsinghua University, Beijing 100084, China; njg23@mails.tsinghua.edu.cn (J.N.); tanyf22@mails.tsinghua.edu.cn (Y.T.); nxn21@mails.tsinghua.edu.cn (X.N.); sxj24@mails.tsinghua.edu.cn (X.S.); celiyang@tsinghua.edu.cn (C.Y.); zhihuili@tsinghua.edu.cn (Z.L.); johnji@tsinghua.edu.cn (J.S.J.); 2 Institute for Healthy China, Tsinghua University, Beijing 100084, China; 3 Oxford Institute of Population Ageing, University of Oxford, Oxford OX1 2JD, UK; yanan.zhang@ageing.ox.ac.uk

**Keywords:** nitrate, sarcopenia, nutrient, diet, UK Biobank, cohort study

## Abstract

Nitrate is abundant in natural foods, especially plant-based foods, having the potential to enhance muscle function. However, its relationship with sarcopenia in the context of daily diet remains unexplored. This cohort study investigated the associations between dietary nitrate intake and sarcopenia, as well as related symptoms, using data including 28,229 participants with a mean follow-up of 9.37 years from the UK Biobank. Dietary nitrate intake was estimated using a comprehensive nitrate food database. Adjusted logistic regression models suggested potential inverse associations between total nitrate intake and risks of sarcopenia plus pre-sarcopenia (Sarc-Presarc), low hand grip strength (HGS), and low walking pace. Similar results were primarily observed for nitrate from plant-based foods. With higher intake, females appeared to have higher HGS and a decreased risk of Sarc-Presarc, while males exhibited a reduced risk of low walking pace. The inverse association between nitrate intake and low skeletal muscle mass index was more evident in individuals aged 65 and above. These associations seemed independent of antioxidants, though higher antioxidants might augment the protective effect against low walking pace. Mediation analyses indicated that protein homeostasis and blood pressure might mediate these associations. These findings suggested that a higher dietary nitrate intake from plant-based foods could contribute to sarcopenia prevention, though further research is needed to confirm these observations.

## 1. Introduction

Sarcopenia is characterized by progressive skeletal muscle deterioration, denoted by co-existing low muscle mass and function [1]. According to guidelines of the European Working Group on Sarcopenia in Older People 2 (EWGSOP2) [2], the global prevalence of sarcopenia was estimated at around 10% [3]. However, projections indicate a significant rise in sarcopenia cases over the next few decades [4], prompting substantial public concern due to its direct links to negative outcomes such as dementia, falls, function decline, frailty, and higher mortality rates [5,6]. The burden of sarcopenia also extends to healthcare costs, with affected individuals incurring significantly higher annual expenses compared to those without sarcopenia [7].

Dietary nitrate, as a natural nutrient, has gained increasing attention due to its potential to promote health [8]. It can be obtained from foods, especially plant-based foods, and converted to nitric oxide (NO) in the body through the nitrate-nitrite-NO pathway [9]. Moreover, previous studies showed that antioxidants might augment this conversion of nitrate to NO [10,11,12]. Skeletal muscles play a key role in nitrate metabolism [13], and NO, in turn, can enhance muscle function [14]. Animal studies have shown that nitrate has the potential to prevent sarcopenia by stimulating muscle regrowth and promoting muscle strength [15,16]. Unfortunately, existing evidence on humans is limited. Previous randomized control trials have suggested that dietary nitrate, particularly from concentrated beetroot juice supplements, may improve muscle power and attenuate muscle damage [17,18,19]. However, these interventions were short-term and immediate (within a week), lacking insight into long-term effects. Additionally, several observational studies showed positive associations between daily dietary nitrate and muscle function in older women and men [20,21]. Yet, prior studies often overlooked muscle mass assessment, a critical factor in diagnosing sarcopenia. These studies failed to reveal the sources of dietary nitrate and the influence of antioxidants on nitrate. Furthermore, a definite mechanism is still lacking for explaining the nitrate-sarcopenia pathways.

Given the established age-related nature of sarcopenia [3], its sex-specific prevalence [2], and the potential effect of antioxidants on nitrate metabolism [10], understanding the independent role of dietary nitrate in mitigating sarcopenia risk and elucidating its mechanism is crucial. To bridge the knowledge gaps, we leveraged prospective data from the UK Biobank to investigate the association between dietary nitrate intake and sarcopenia risk, adjusting possible confounding factors. Additionally, we explored mediation pathways involving specific indicators. These findings aim to provide updated insights into strategies for sarcopenia prevention and muscle health promotion.

## 2. Method

### 2.1. Study Design and Participants

This prospective cohort study utilized data from the UK Biobank, a population-based, large-scale biomedical database encompassing participants from England, Wales, and Scotland. Detailed information regarding the UK Biobank’s structure and methodology has been previously documented [22]. In essence, the UK Biobank recruited over half a million individuals aged 40–69 years residing in the community to attend the baseline assessment between 2006 and 2010, followed by a repeat assessment between 2012 and 2013 and four online visits between 2009 and 2012. Subsequently, the UK Biobank conducted two additional follow-ups after 2014. Based on the study objectives, the current design encompassed participants who provided 24-h dietary recalls at baseline and underwent physical examinations during follow-up.

**Figure 1:**
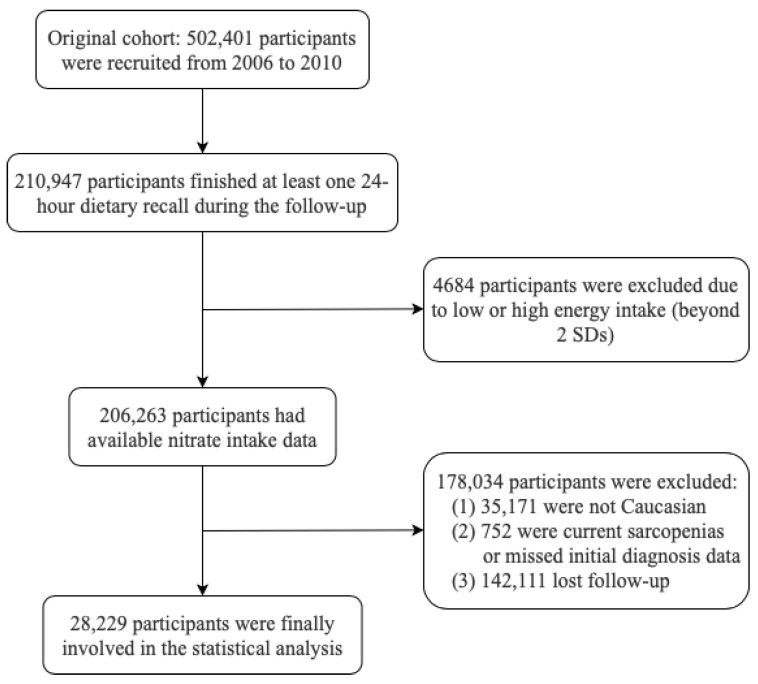
Flowchart of inclusion and exclusion (Abbreviation: SD, standard difference).

The flowchart depicts the inclusion and exclusion criteria (Figure 1). Among 210,947 participants who reported at least one 24-h dietary recall, 4684 were excluded due to extremely low or high energy intake (beyond 2 standard deviations, i.e., energy intake < 614.11 kcal or >5242.76 kcal). As the method used in sarcopenia assessment—the Janssen equation—was only validated for Caucasian ethnicity [23], 35,171 non-Caucasian participants were excluded. Additionally, to avoid reverse causality, 752 individuals were excluded due to existing sarcopenia or missing information for sarcopenia assessment at baseline. Up to 2022, 142,111 individuals were further excluded due to incomplete follow-up. Ultimately, a total of 28,229 participants were included in the analysis.

### 2.2. Estimation of Dietary Nitrate Intake

The 24-h dietary recall was conducted using a self-administered Oxford WebQ questionnaire comprising over 200 food items with standardized portion sizes, which is validated and widely used in large cohort studies [24]. The average nitrate was calculated based on multiple 24-h dietary recalls from recruitment to 2012, assuming that the average value could represent an even nitrate intake level over the long term.

A comprehensive food nitrate database was utilized [25,26] to determine dietary nitrate content, developed through a systematic review, and widely adopted for estimating food nitrate intake [27,28]. However, due to the absence of a specific nitrate database for beverages, the nitrate content of drinking water was approximated using the median level (0.727 mg/L) from a survey of bottled mineral waters in the British Isles [29]. Nitrate intakes from alcoholic beverages, beverage products, mixed foods (e.g., pizza and snacks), vegetarian alternatives, oils, and condiments were not calculated due to unknown ingredients and proportions in these complex foods. Similarly, since the Oxford WebQ questionnaire did not collect specific information on cooking methods, nitrate losses during the cooking were not accounted for. However, the food nitrate database utilized in this study comprised 158 plant- and animal-based food types, nearly covering participants’ dietary spectrum in the UK Biobank, thereby enabling the estimation of their daily dietary nitrate intake levels to the fullest extent possible. Specifically, nitrate intake was calculated by multiplying the food portion size by the weight per serving and the median value of the corresponding food nitrate content. The resulting unit of measurement was saved as milligrams (mg).

$$Dietary\;Nitrate\;Intake\left(mg\right)=\sum_{i=1}^{n}\left(A_{i}\times B_{i}\times C_{i}\right)$$

*A*: Food Portion Size; *B*: Weight per Portion; *C*: Median Food Nitrate Content

*n*: Total Number of Foods; *i*: Specific food number

Water for nitrate estimation encompassed drinking water, tea, and coffee. For food items not included in the food nitrate database, the nitrate value was substituted with the average nitrate value of the similar food type or the corresponding food group. For example, berries were replaced by strawberries and shellfish in the seafood group. Furthermore, the food nitrate database was established based on fresh weight as a standard, while several foods in the Oxford WebQ questionnaire were diluted or concentrated; considering that these processes might influence the estimation of nitrate [30], we supposed an approximate nitrate content change rate of 0.5 for soups and juices and 1.5 for dried fruits to minimize the estimation bias as much as possible. For example, the nitrate content in orange juice was estimated to be half that of fresh orange, while the nitrate content in prune was estimated to be 1.5 times that of fresh plum.

### 2.3. Assessment of Sarcopenia and Related Symptoms

Sarcopenia was diagnosed based on co-existing low hand grip strength (HGS) and low skeletal muscle mass index (SMI) according to EWGSOP2 [2], and pre-sarcopenia was considered as the status of single low HGS or single low SMI [31]. We defined sarcopenia plus pre-sarcopenia (Sarc-Presarc) as a secondary outcome. Within the UK Biobank, HGS was measured using a Jamar J00105 hydraulic hand dynamometer, with low HGS defined by cut-off values of <27 kg for men and <16 kg for women, respectively. SMI was computed as appendicular skeletal muscle mass (ASM) divided by the square of height. ASM was derived using the Janssen equation [23], utilizing impedance measured by a Tanita BC418MA body composition analyzer. Low SMI was determined by cut-off values of <7.0 kg/m^2^ for men and <5.5 kg/m^2^ for women, respectively.

Sarcopenia combined with low physical performance ought to be further diagnosed as severe sarcopenia [2]. However, since the usual walking pace data was self-reported in the UK Biobank and its cut-off point was higher (<1.3 m/s) compared to that in EWGSOP2 guidelines (≤0.8 m/s), assessment of low physical performance and subsequent diagnosis of severe sarcopenia were not supported. Nonetheless, self-reported low walking pace as a distinct outcome was still included in the analysis.

### 2.4. Other Variables

Baseline characteristics that were considered to have potential confounding effects on the association between dietary nitrate intake and sarcopenia were gathered through a structured questionnaire, encompassing various sociodemographic factors including sex, age, body mass index, educational attainment, and Townsend deprivation index; lifestyles including smoking status, drinking status, and physical activity level; cardiovascular disease (CVD) status; follow-up duration (years from baseline to the final visit); and dietary nutrient intake data including total energy, carbohydrates, protein, fat, and composite dietary antioxidant index (CDAI).

Specifically, the Townsend deprivation index is a composite measure based on unemployment, non-car ownership, non-home ownership, and household overcrowding, serving as a socioeconomic gauge reflecting relative privation [32]. Physical activity levels were categorized based on the International Physical Activity Questionnaire [33]. Self-reported CVD status was utilized to assess the usage of nitrate medicines like nitroglycerin and isosorbide mononitrate since these drugs are commonly prescribed for CVD management and may have similar effects to nitrate. Dietary nutrient intake assessments were conducted using Oxford WebQ’s built-in algorithms and food composition data. Additionally, CDAI was calculated as the sum of standardized values of dietary vitamin A retinal equivalents, vitamin C, vitamin E, zinc, selenium, and manganese, serving as a widely accepted metric for reflecting dietary antioxidant levels, with higher index values indicating greater antioxidant capacity [34].

Data from repeated measured physical examinations and blood assays (from baseline to the first follow-up) were integrated as average values and examined as potential mediators to elucidate their roles in the relationship between dietary nitrate intake and sarcopenia. These indicators encompassed inflammatory biomarkers including white blood cell count, neutrophil count, serum alkaline phosphatase, and serum C-reactive protein; serum protein metabolomes including total protein, albumin, and creatinine; serum lipid metabolomes including apolipoprotein A, apolipoprotein B, high-density lipoprotein, low-density lipoprotein, cholesterol, lipoprotein A, and triglycerides; serum hormone metabolomes including insulin-like growth factor-1, estradiol, testosterone, and sex home-binding globulin; as well as blood pressure parameters including systolic pressure and diastolic pressure.

### 2.5. Statistical Analysis

Dietary nitrate intake was categorized into four levels (Q_1_, Q_2_, Q_3_, and Q_4_) based on the median and quartile due to its skewed distribution. In the baseline characteristics, continuous variables with a normal distribution were presented as mean ± standard deviation and assessed using the linear trend test. Continuous variables with a skew distribution were displayed as median [*P*_25_, *P*_75_] and analyzed using Spearman’s rank correlation. Categorical variables were presented as numbers (percentage) and tested using the trend chi-square test for ordered variables or Pearson’s chi-square test for unordered variables.

Four regression models were constructed to evaluate associations of dietary nitrate intake levels with sarcopenia and related symptoms. Model A adjusted for sociodemographic factors and lifestyle variables to mitigate potential confounding. Model B, built upon Model A, included adjustments for CVD status and follow-up duration to account for the impacts of medication and observation time. Model C further adjusted for dietary energy, carbohydrates, protein, and fat intake based on Model B to control for dietary influences. Finally, Model D additionally incorporated adjustment for CDAI from Model C to consider the impact of dietary antioxidants. Missing covariate values were imputed using multiple imputations with a random forest method under the assumption of missingness at random.

Logistic regression analyses were utilized for binary outcomes, including sarcopenia, Sarc-Presarc, low HGS, low SMI, and low walking pace, yielding odds ratios (ORs) with 95% confidence intervals (CIs). Linear regression analyses were further employed for specific sarcopenia-related parameters, including values of the last HGS, SMI, and ASM and values of Δ HGS, Δ SMI, and Δ ASM, providing *β* coefficients with 95% CIs. Subgroup analyses were conducted based on different sources of dietary nitrate, exploring variations in results across dietary nitrate intake levels from plant-based foods, animal-based foods, and water.

Stratified analyses were performed to investigate differences in associations between dietary nitrate intake levels and outcomes across different sex and age groups. The age was divided into two groups based on the cut-off point of 65 years, beyond which is a risk factor for sarcopenia [35]. Additionally, a stratified analysis was conducted on CDAI to determine the potential influence of antioxidants on nitrate. These stratification analyses were based on Model D, and the regression model did not adjust for the corresponding stratification factor.

We assumed that dietary nitrate intake may influence specific biomarkers, which, in turn, affect sarcopenia development. Therefore, regression-based mediation analyses were performed utilizing the previously identified potential mediators to examine whether these indicators can mediate the association between dietary nitrate intake and sarcopenia. Mediation analysis can portion the total effect into direct and indirect components, and the primary results are the latter, including the average causal mediation effect (ACME) and the mediation proportion, which can explain the role of these factors on observed associations. All mediation analyses were based on Model D.

Sensitivity analyses were conducted to assess the stability and consistency of results. Data without imputation were analyzed in the same manner for comparison with imputed results. Additionally, a reanalysis was performed after excluding individuals with a follow-up duration of less than 5 years.

Trend tests were performed alongside all regression models. Statistical analyses were carried out using R (version 4.2.2), with “mice” and “mediation” packages employed for imputation and mediation analyses, respectively. A significance level of *p* < 0.05 (two-tailed) was considered statistically significant.

## 3. Results

### 3.1. Distribution of Dietary Nitrate

**Figure 2:**
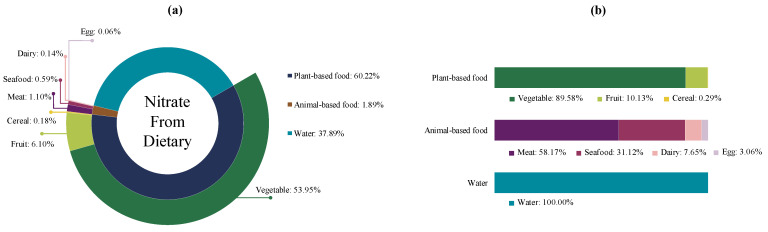
Sources of dietary nitrate. (**a**) Proportion of dietary nitrate from different foods. (**b**) Proportion of dietary nitrate in different food groups.

The median [*P*_25_, *P*_75_] of raw daily dietary nitrate among our participants was 144.40 [77.07, 247.82] mg. Figure 2 presents the sources of dietary nitrate among participants in this study. Daily dietary nitrate was sourced from vegetables (53.95%), water (37.89%), fruits (6.10%), meats (1.10%), seafood (0.59%), cereals (0.18%), dairy (0.14%), and eggs (0.06%) in order. In total, nearly three-quarters of dietary nitrate was derived from plant-based foods, which were mainly vegetables (89.58%). However, only a small proportion (1.89%) of nitrate was constituted from animal-based foods, which were mainly meat (58.17%) and seafood (31.12%). A total of 37.89% of nitrate was derived from water.

### 3.2. Study Population Characteristics

A total of 28,229 participants were included in the analysis, with a mean follow-up duration of 9.37 years. Table 1 presents a detailed summary of participant characteristics grouped by quartiles of dietary nitrate intake. Females accounted for 51.7% of the cohort, with a higher portion in the high nitrate intake group compared to men. The average age of the participants was 55 years old, and there was a slight positive correlation between age and nitrate intake levels. An inverse trend was demonstrated between nitrate levels and body mass index. Educational levels varied significantly across the quartiles of nitrate intake, with a higher proportion of participants with college/university qualifications observed in groups with higher nitrate consumption.

Smoking status showed a negative trend with nitrate intake levels, whereas physical activity levels exhibited a positive correlation. Additionally, participants with higher dietary nitrate intake consumed more energy, carbohydrates, protein, and fat and displayed higher CDAI values. However, no significant differences in the Townsend deprivation index, drinking status, CVD status, and follow-up time were found across nitrate intake levels.

### 3.3. Sarcopenia and Related Symptoms

Only 0.6% of participants developed new-onset sarcopenia, while 12.1% experienced Sarc-Presarc. Among these individuals, 6.2% and 6.4% suffered from low HGS and low SMI, respectively. Additionally, 4.7% of participants reported a low walking pace. As shown in Supplemental Table S1, when no covariates were adjusted, different levels of nitrate intake appeared to be associated with the incidence rates of low HGS, low walking pace inversely, and low SMI positively. No significant associations of nitrate intake levels with the incidence rates of sarcopenia and Sarc-Presarc were observed. Furthermore, among sarcopenia-related parameters, nitrate intake levels appeared to demonstrate negative correlations with values of the last HGS, SMI, and ASM.

### 3.4. Associations Between Nitrate Intake Levels and the Risk of Sarcopenia

After adjusting potential confounders, we observed significant negative associations of nitrate intake levels with the risks of Sarc-Presarc, low HGS, and low walking pace across various models, as well as significant dose-response trends. As presented in Table 2, for low SMI, we only observed a significant association in Model B, compared with the Q_1_ group, the Q_4_ group had a 20% lower risk of low SMI (OR = 0.80, 95% CI: 0.68, 0.94); however, after adjusting for dietary nutrients and CDAI, this significance dissipated. Nonetheless, no significant associations were found between dietary nitrate intake levels and sarcopenia across all four models.

**Figure 3:**
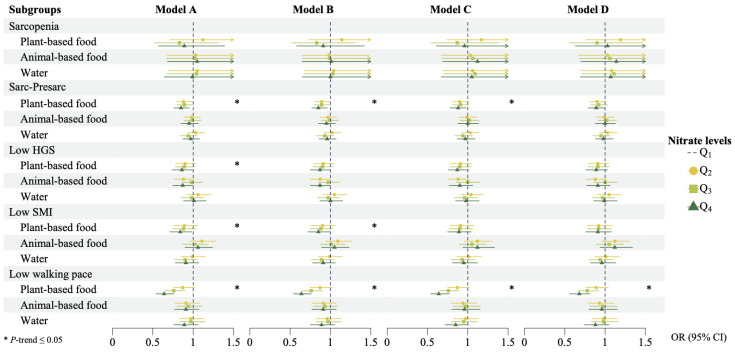
Differences in regression results of dietary nitrate from different sources (Abbreviation: CI, confidence interval; HGS, hand grip strength; OR, odds ratio; Sarc-Presarc, sarcopenia plus pre-sarcopenia; SMI, skeletal muscle mass index).

A subgroup analysis for the different sources of dietary nitrate was further conducted, and the results are depicted in Figure 3. After adjusting for all considered covariates in Model D, only nitrate from plant-based foods exhibited significant negative associations with Sarc-Presarc and low walking pace. No similar associations were observed for nitrate from animal-based foods and water.

The results of linear regression and subgroup analysis for continuous outcomes are outlined in Supplemental Table S2 and Supplemental Figure S1, respectively. In summary, after adjusting for covariates in Model D, no significant associations were found between total nitrate and values of the last HGS, SMI, and ASM. However, in the subgroup analysis, nitrate from plant-based foods displayed a positive correlation with the value of the last ASM. Specifically, the ASM values of the Q_3_ and Q_4_ groups were 0.12 (95% CI: 0.02, 0.21) and 0.13 (95% CI: 0.03, 0.23) kg higher than that of the Q_1_ group, respectively.

### 3.5. Stratified Analysis by Sex, Age, and CDAI

**Figure 4:**
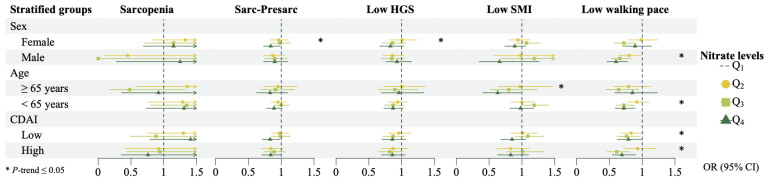
Differences in regression results of different sex, age, and CDAI groups (Abbreviation: CDAI, composite dietary antioxidant index; CI, confidence interval; HGS, hand grip strength; OR, odds ratio; Sarc-Presarc, sarcopenia plus pre-sarcopenia; SMI, skeletal muscle mass index).

Considering that sex, age, and CDAI may have a potential influence on nitrate metabolism, we conducted stratified analyses to identify the differences in the associations among different subgroups. Figure 4 lists the results of stratified analyses for the associations of nitrate intake with sarcopenia, Sarc-Presarc, low HGS, low SMI, and low walking pace. Varying associations were found in different sex groups. High nitrate intake was significantly inversely associated with the risks of Sarc-Presarc and low HGS in females, while no significant associations were observed in males. Conversely, dietary nitrate was significantly negatively associated with the risk of low walking pace in males but not in females. Moreover, we identified a potential age-related impact on the association between dietary nitrate and low walking pace. Nitrate intake levels were significantly negatively associated with low SMI among participants ≥ 65 years old, whereas this association was not significant among those < 65 years old. Meanwhile, there was a significant dose-response trend in the negative association between dietary nitrate and low walking pace only among people < 65 years old. Furthermore, a high level of CDAI seemed to enhance the effect of nitrate. High nitrate intake was significantly associated with a reduced risk of Sarc-Presarc both in low and high CDAI groups. Intriguingly, among participants consuming high nitrate, individuals with high CDAI had a lower risk of low walking pace than those with low CDAI.

The results of stratified analyses for sarcopenia-related parameters are detailed in Supplemental Figure S2. In summary, high nitrate intake significantly increased the HGS values in females, not in males. Nonetheless, no significant results were observed for other parameters in different subgroups.

### 3.6. Mediation Effects

We conducted mediation analyses for three outcomes, including sarcopenia, low HGS, and low walking pace, which were significantly associated with nitrate intake levels in Model D. The mediation analysis results are presented in Table 3. Notably, serum creatinine emerged as a consistent mediator with significant ACME for the associations of dietary nitrate with Sarc-Presarc, with the mediation proportion accounting for −6.48% of the total effect. Serum albumin served as a significant mediator for the associations of dietary nitrate with low HGS and low walking pace, with mediation proportions accounting for 4.14% and 1.53%, respectively. Additionally, systolic pressure was identified as a potential mediator for the associations of nitrate with Sarc-Presarc and low HGS, accounting for 3.82% and 2.06% of mediation proportions, respectively. Moreover, diastolic pressure also mediated the association between nitrate and Sarc-Presarc (3.37% of mediation proportion). However, no mediation effects were observed for other indicators.

### 3.7. Sensitivity Analysis Results

No significant alternations were observed in the baseline characteristics of participants following imputation, as depicted in Supplemental Table S3. Regression results were similar between before and after imputation. Moreover, after excluding individuals with a follow-up duration of less than 5 years, the regression results remained stable. Detailed sensitivity analysis results are illustrated in Supplemental Figures S3 and S4.

## 4. Discussion

To the best of our knowledge, this prospective cohort study is the first large-scale investigation into the association between dietary nitrate and sarcopenia, considering stratifications by sex, age, and CDAI and exploring the potential mediation effects of twenty indicators. We found that higher dietary nitrate intake, particularly from plant-based sources, was associated with significantly reduced risks of certain sarcopenia-related symptoms. These associations varied by sex, age, and CDAI. Additionally, our results suggest that the pathways of nitrate against sarcopenia development may involve the regulation of protein metabolism and blood pressure.

The risks and benefits of nitrate remain under ongoing investigation and debate. Excessive intake of nitrate, particularly from processed meats, may contribute to the formation of N-nitrosamines, potential carcinogens associated with cancer risk [36]. However, long-term nitrate intake at doses similar to those in Western diets does not appear detrimental [37]. Moreover, dietary nitrate has also been linked to potential health benefits, such as preventive effects on dementia and cardiovascular disease [27,28]. The European Food Safety Authority recommends an acceptable daily intake of nitrate ranging from 0 to 3.7 mg/kg body weight [38], and the vast majority (88.2%; not shown in the Results) of participants in our cohort consumed nitrate within this recommended. Furthermore, different cooking methods can reduce actual nitrate consumption [30], suggesting that the nitrate dose from a daily diet is appropriate for most people.

Nutritional supplements have been theorized to potentially manage sarcopenia. However, the direct relationship between dietary nitrate intake and the risk of sarcopenia has not been extensively studied. Previous studies have reported that nitrate supplements may enhance exercise performance in both young athletes and older adults [17,18,19], although these effects were observed in short-term interventions. The long-term implications of daily dietary nitrate intake for chronic conditions such as sarcopenia are of greater public health significance. Only two observational studies in the Australian population suggested that dietary nitrate might increase muscle power and walking speed [20,21], which is consistent with some of our findings. A novelty in our study is the assessment of muscle mass alongside muscle function, allowing for a more comprehensive evaluation of the association between dietary nitrate and sarcopenia. We did not find significant results for diagnosed sarcopenia, possibly due to rare new-onset definite cases (0.6%). However, we observed significant associations of nitrate with lower risks of Sarc-Presarc, low HGS, and low walking pace, which can provide a similar perspective.

The source of nitrate should be considered. Previous evidence indicates that nitrate from plant-based foods is safer compared to nitrate from animal-based foods [39]. In our study, we observed significant associations between nitrate from plant-based foods and reduced risks of sarcopenia-related symptoms, while no such associations were found for nitrate from animal-based foods and water. This may be because plant-based foods are rich in nitrate as well as other beneficial components like antioxidants. Conversely, animal-based foods, especially red meat, contain little nitrate but are high in unhealthy substances such as saturated fats. Moreover, Yoo et al. reported a link between inadequate dietary water intake and sarcopenia in older Korean adults [40], a finding we did not replicate, possibly due to the higher median nitrate content in South Korean drinking water compared to British water (3.0 vs. 0.7 mg/L) [41]. Given that dietary nitrate is mainly derived from plants, especially leaf and stem vegetables [25], our study highlights the importance of consuming nitrate predominantly from plant-based sources.

We further explored the influence of antioxidants on nitrate. Antioxidants are generally considered beneficial for preventing sarcopenia. Notably, they are vital for producing NO since the conversion of nitrate to NO can be reversed by oxidation [9]. Prior animal studies have reported that zinc and manganese are linked to increasing NO production [42,43]. Moreover, several clinical trials have shown that β-carotene, vitamin C, vitamin E, and selenium can promote NO production in humans [10,11,12,44]. Hence, we suppose that antioxidants can enhance the effects of dietary nitrate by ensuring the nitrate-nitrite-NO pathway. However, previous studies have not adequately clarified the roles of antioxidants and nitrate in sarcopenia development. Our findings from regression Model D, which was additionally adjusted for CDAI, suggest that dietary nitrate independently protects against sarcopenia-related symptoms. We found a protective effect of high nitrate intake on Sarc-Presarc and low walking pace in both low and high CDAI groups, with a stronger impact on the latter symptom in the high CDAI group. These results support our subgroup analysis, indicating that nitrate from plant-based foods, rich in antioxidants such as vitamin C and β-carotene, is beneficial in preventing sarcopenia.

We also examined the differences in associations of dietary nitrate with sarcopenia-related symptoms based on sex and age. In sex-based analyses, the association between dietary nitrate and Sarc-Presarc was particularly significant among females, which may be due to females having a stronger ability to convert nitrate to nitrite in the entero-salivary circulation compared to males [45]. Conversely, males benefited more from nitrate in terms of improving low walking pace, similar to prior findings on athletes’ physical performance [46]. This may be explained by sex-based differences in skeletal muscle kinetics and fiber-type composition [47]. In age-based analyses, we observed a significant association between high nitrate intake and a reduced risk of low SMI in older adults but not in younger ones. This finding is intriguing since aging is generally associated with declines in NO bioavailability [48]. Yet, Piknova et al. also observed that old rats could accumulate more nitrate in their organs than young rats after nitrate supplementing [13], supporting the hypothesis that sufficient nitrate storage might alleviate the impact of declined NO bioavailability. Other studies did not find significant differences in the effects of nitrate on muscle function or physical performance by sex and age [21,49], indicating that more evidence is needed for precise nutritional guidance.

The exact biological mechanisms through which dietary nitrate influences the occurrence of sarcopenia remain elusive. Existing evidence suggests that inflammation [50], disrupted protein metabolism [51], dyslipidemia [52], hormonal changes [53], and systemic vascular function [54] may all contribute to the development of sarcopenia. Our mediation analyses indicate that increased serum albumin and reduced serum creatinine, biomarkers of protein metabolism, are the key mediators in the association between dietary nitrate intake and muscle health. Serum albumin is positively related to muscle strength and mass [55,56], while serum creatinine can reflect muscle catabolism conditions [57]. Petrick et al. also found that dietary nitrate might preserve the mitochondrial protein synthesis of mice [58]. Therefore, the effects of nitrate on muscle health may be mediated by maintaining muscle protein homeostasis. Additionally, we found that increasing blood pressure parameters, including systolic and diastolic pressure, demonstrated mediating effects on the associations of nitrate with sarcopenia-related symptoms. Taekema et al. and Blanchard et al. reported that higher blood pressure was linked to higher muscle strength [59,60], which partly explains our findings. However, this seems contradictory to previous research since nitrate tends to decrease blood pressure by producing NO [61,62]. Due to the limited evidence, more in-depth studies are urgently needed to reveal the exact role of blood pressure. Furthermore, we did not observe any significant mediating effects of inflammation biomarkers, serum lipid metabolomes, and hormone metabolomes. The concrete pathways linking dietary nitrate to sarcopenia remain to be confirmed in future in-depth studies.

This study boasts several strengths. Firstly, it was based on a large-scale British database, providing adequate statistical power and public health relevance. Secondly, we utilized a comprehensive nitrate food database to estimate nitrate intake from plant- and animal-based foods and water, minimizing potential misclassification bias. Thirdly, we further considered the influence of antioxidants on nitrate, offering new insights into the independent role of nitrate in sarcopenia development. Finally, sensitivity analyses were conducted to ensure the robustness of our results.

However, several limitations of this study must be acknowledged. Firstly, although our results are similar to studies on other populations, such as Americans and Australians, the generalizability of our findings to other ethnic populations may still be limited due to variations in genetic backgrounds and dietary habits. Secondly, reliance on 24-h dietary recalls introduces potential recall and reporting biases, and changes in food choices over time were not fully captured. Thirdly, assessing the precise dosage of dietary nitrate was challenging due to missing cooking method information and the exclusion of complex foods, which could impact the accuracy of nitrate consumption estimates. Fourthly, the UK Biobank’s incomplete physical examination data for all participants and loss of follow-up information introduce potential selection biases. Lastly, despite adjusting for numerous confounders, residual confounding may persist, warranting caution in interpreting the study findings.

## 5. Conclusions

Our long-term follow-up study has yielded insights into the associations between higher dietary nitrate intake and reduced risks of sarcopenia-related outcomes, which may vary by sex and age. Nitrate from plant-based foods plays a prominent role in these associations. The protective effects of dietary nitrate on sarcopenia development are independent of antioxidants, although antioxidants may enhance the effects of nitrate. These associations may be mediated by muscle protein metabolism and blood pressure regulation. Our findings highlight potential strategies for preventing and managing sarcopenia through dietary interventions. Moving forward, further studies are warranted to validate our findings in different populations, establish precise guidelines for safe and effective nitrate consumption, and elucidate the underlying mechanisms linking dietary nitrate to the risk of sarcopenia.

## Figures and Tables

**Table 1:** Characteristics of participants among different dietary nitrate intake level groups.

Characteristics ^1^	Overall	Dietary Nitrate Intake Levels Grouped by Quartiles (mg) ^3^				
		Q_1_ (0.43–77.07)	Q_2_ (77.07–144.30)	Q_3_ (144.30–247.82)	Q_4_ (247.84–2306.38)	*p*
Sex						<0.001
Female	14,601 (51.7%)	3141 (44.5%)	3551 (50.3%)	3896 (55.2%)	4013 (56.9%)	
Male	13,628 (48.3%)	3916 (55.5%)	3506 (49.7%)	3161 (44.8%)	3045 (43.1%)	
Age (year)	55.47 ± 7.53	54.95 ± 7.53	55.67 ± 7.48	55.57 ± 7.48	55.70 ± 7.59	<0.001
Body mass index (kg/m^2^)	26.47 ± 4.21	26.77 ± 4.21	26.48 ± 4.22	26.34 ± 4.20	26.28 ± 4.20	<0.001
Missing	17 (0.1%)	2 (0.0%)	6 (0.1%)	3 (0.0%)	6 (0.1%)	
Educational levels ^2^						
College/University	13,729 (48.6%)	3036 (43.0%)	3408 (48.3%)	3525 (50.0%)	3760 (53.3%)	<0.001
A/AS levels	3837 (13.6%)	939 (13.3%)	979 (13.9%)	959 (13.6%)	960 (13.6%)	
O levels/GCSEs	5377 (19.0%)	1495 (21. 2%)	1337 (18.9%)	1327 (18.8%)	1218 (17.3%)	
CSEs	1007 (3.6%)	341 (4.8%)	250 (3.5%)	225 (3.2%)	191 (2.7%)	
NVQ/HND/HNC	1473 (5.2%)	461 (6.5%)	373 (5.3%)	354 (5.0%)	285 (4.0%)	
Other qualifications	1283 (4.5%)	298 (4.2%)	310 (4.4%)	323 (4.6%)	352 (5.0%)	
None of the above	1473 (5.2%)	470 (6.7%)	388 (5.5%)	331 (4.7%)	284 (4.0%)	
Missing	50 (0.2%)	17 (0.2%)	12 (0.2%)	13 (0.2%)	8 (0.1%)	
Townsend deprivation index	−2.04 ± 2.62	−2.00 ± 2.64	−2.11 ± 2.57	−2.06 ± 2.62	−1.99 ± 2.64	0.720
Missing	26 (0.1%)	6 (0.1%)	9 (0.1%)	5 (0.1%)	6 (0.1%)	
Smoking status						<0.001
Never	17,256 (61.1%)	4238 (60.1%)	4313 (61.1%)	4350 (61.6%)	4355 (61.7%)	
Previous	9387 (33.3%)	2314 (32.8%)	2374 (33.6%)	2333 (33.1%)	2366 (33.5%)	
Current	1529 (5.4%)	494 (7.0%)	360 (5.1%)	357 (5.1%)	318 (4.5%)	
Missing	50 (0.2%)	11 (0.2%)	10 (0.1%)	17 (0.2%)	19 (0.3%)	
Drinking status						0.064
Never	563 (2.0%)	124 (1.8%)	138 (2.0%)	136 (1.9%)	165 (2.3%)	
Previous	587 (2.1%)	159 (2.3%)	135 (1.9%)	146 (2.1%)	147 (2.1%)	
Current	27,074 (95.9%)	6773 (96.0%)	6781 (96.1%)	6774 (96.0%)	6746 (95.6%)	
Missing	5 (0.0%)	1 (0.0%)	3 (0.0%)	1 (0.0%)	0 (0.0%)	
Physical activity						<0.001
Low level	4466 (15.8%)	1315 (18.6%)	1131 (16.0%)	1059 (15.0%)	961 (13.6%)	
Moderate level	10,505 (37.2%)	2588 (36.7%)	2695 (38.2%)	2646 (37.5%)	2576 (36.5%)	
High level	9637 (34.1%)	2175 (30.8%)	2301 (32.6%)	2512 (35.6%)	2649 (37.5%)	
Missing	3621 (12.8%)	979 (13.9%)	930 (13.2%)	840 (11.9%)	872 (12.4%)	
CVD status						0.054
No	22,083 (78.2%)	5463 (77.4%)	5494 (77.9%)	5596 (79.3%)	5530 (78.4%)	
Yes	6125 (21.7%)	1587 (22.5%)	1561 (22.1%)	1453 (20.6%)	1524 (21.6%)	
Missing	21 (0.1%)	7 (0.1%)	2 (0.0%)	8 (0.1%)	4 (0.1%)	
Follow-up time (year)	9.37 ± 2.17	9.37 ± 2.18	9.35 ± 2.15	9.36 ± 2.18	9.39 ± 2.18	0.628
Nutrients intake						
Energy (kcal)	2029.34 [1719.82, 2381.67]	1932.74 [1609.76, 2285.42]	2022.80 [1724.83, 2369.85]	2044.52 [1752.37, 2389.89]	2111.31 [1797.29, 2478.95]	<0.001
Carbohydrates (g)	249.72 [207.38, 296.07]	235.07 [192.11, 279.86]	244.83 [205.95, 289.45]	251.88 [210.68, 296.56]	267.56 [223.41, 314.67]	<0.001
Protein (g)	79.42 [66.83, 93.20]	75.54 [62.31, 89.65]	79.96 [67.96, 93.05]	80.50 [68.23, 93.70]	81.53 [68.53, 95.97]	<0.001
Fat (g)	70.47 [55.22, 88.08]	66.20 [50.91, 84.28]	71.13 [55.93, 88.55]	71.68 [57.01, 88.30]	72.76 [57.28, 91.01]	<0.001
CDAI	−0.30 [−2.51, 2.15]	−2.03 [−4.13, 0.24]	−0.48 [−2.46, 1.65]	0.14 [−1.90, 2.44]	1.17 [−1.10, 3.78]	<0.001

Abbreviations: CDAI, composite dietary antioxidant index; CVD, cardiac vascular disease. ^1^ Categorical variables were shown as numbers (percentage) and tested using the trend chi-square test for the ordered variables or the chi-square test for the unordered variables; continuous variables with normal distribution were shown as mean ± standard deviation and tested using the linear trend test; continuous variables with skew distribution were shown as median [*P*_25_, *P*_75_] and tested using Spearman rank correlation. ^2^ Each item involved equivalent levels as described. “Other qualifications” meant other professional qualifications such as nursing and teaching. ^3^ Dietary nitrate was categorized into four levels (Q_1_, Q_2_, Q_3_, and Q_4_) based on the median and quartile.

**Table 2:** Associations of dietary nitrate with sarcopenia and related symptoms.

**Nitrate Levels ^1^**	**Sarcopenia**	**Sarc-Presarc**	**Low HGS**	**Low SMI**	**Low Walking Pace**
	**OR (95% CI)**				
Q_1_	1.00 (Reference)	1.00 (Reference)	1.00 (Reference)	1.00 (Reference)	1.00 (Reference)
Model A ^2^					
Q_2_	1.11 (0.72, 1.73)	0.90 (0.81, 1.00)	0.91 (0.80, 1.04)	0.91 (0.78, 1.07)	0.86 (0.74, 1.00)
Q_3_	0.90 (0.57, 1.42)	0.92 (0.83, 1.02)	**0.84 (0.73, 0.96)**	1.01 (0.86, 1.18)	**0.67 (0.57, 0.80)**
Q_4_	0.96 (0.61, 1.49)	**0.83 (0.74, 0.93)**	**0.87 (0.75, 0.99)**	**0.80 (0.68, 0.94)**	**0.72 (0.61, 0.85)**
*P*-trend	0.607	0.002	0.021	0.029	<0.001
Model B ^3^					
Q_2_	1.14 (0.73, 1.78)	0.91 (0.81, 1.01)	0.92 (0.80, 1.05)	0.92 (0.78, 1.08)	0.86 (0.74, 1.01)
Q_3_	0.91 (0.58, 1.44)	0.93 (0.83, 1.03)	**0.84 (0.73, 0.97)**	1.02 (0.87, 1.19)	**0.67 (0.57, 0.79)**
Q_4_	0.96 (0.62, 1.51)	**0.83 (0.74, 0.92)**	**0.87 (0.75, 1.00)**	**0.80 (0.68, 0.94)**	**0.72 (0.61, 0.85)**
*P*-trend	0.608	0.002	0.021	0.028	<0.001
Model C ^4^					
Q_2_	1.21 (0.77, 1.88)	0.92 (0.82, 1.02)	0.92 (0.80, 1.06)	0.94 (0.80, 1.11)	0.86 (0.73, 1.00)
Q_3_	0.97 (0.61, 1.54)	0.94 (0.84, 1.05)	**0.84 (0.73, 0.97)**	1.06 (0.90, 1.24)	**0.66 (0.56, 0.79)**
Q_4_	1.06 (0.67, 1.68)	**0.84 (0.75, 0.95)**	**0.86 (0.75, 0.99)**	0.85 (0.72, 1.00)	**0.70 (0.60, 0.83)**
*P*-trend	0.919	0.008	0.019	0.165	<0.001
Model D ^5^					
Q_2_	1.24 (0.79, 1.94)	0.92 (0.83, 1.03)	0.92 (0.81, 1.06)	0.95 (0.81, 1.12)	0.87 (0.75, 1.02)
Q_3_	1.02 (0.64, 1.63)	0.95 (0.85, 1.06)	**0.85 (0.73, 0.98)**	1.07 (0.91, 1.26)	**0.69 (0.58, 0.82)**
Q_4_	1.14 (0.71, 1.85)	**0.85 (0.76, 0.96)**	0.87 (0.75, 1.01)	0.87 (0.73, 1.03)	**0.74 (0.62, 0.88)**
*P*-trend	0.847	0.021	0.036	0.289	<0.001

Abbreviations: CI, confidence interval; HGS, hand grip strength; OR, odds ratio; Sarc-Presarc, sarcopenia plus pre-sarcopenia; SMI, skeletal muscle mass index. Bold: *p* < 0.05. ^1^ Dietary nitrate was categorized into four levels (Q_1_, Q_2_, Q_3_, and Q_4_) based on the median and quartile. ^2^ Model A adjusted for covariates including sex, age, body mass index, educational levels, Townsend deprivation index, smoking status, drinking status, and physical activity levels. ^3^ Model B adjusted for CVD status and follow-up duration based on Model A. ^4^ Model C adjusted for nutrient intakes based on Model B, including dietary energy, carbohydrates, protein, and fat. ^5^ Model D adjusted for CDAI based on Model C.

**Table 3:** Mediation effects of certain indicators in associations of dietary nitrate with Sarc-Presarc, low HGS, and low walking pace.

Mediator	Sarc-Presarc		Low HGS		Low Waking Pace	
	ACME	m%	ACME	m%	ACME	m%
Inflammation biomarkers						
White blood cell count	−3.84 × 10^−5^	0.86	−1.86 × 10^−5^	0.62	−1.15 × 10^−5^	0.21
Neutrophil count	−4.21 × 10^−5^	0.96	−3.00 × 10^−5^	1.03	−1.61 × 10^−5^	0.29
Serum alkaline phosphatase	−5.43 × 10^−4^	1.29	−1.40 × 10^−5^	0.48	−8.63 × 10^−6^	0.15
Serum C-reactive protein	−4.22 × 10^−5^	0.99	−1.92 × 10^−5^	0.66	−1.06 × 10^−5^	0.18
Serum protein metabolomes						
Total protein	−1.48 × 10^−5^	0.44	−5.57 × 10^−6^	0.25	−3.09 × 10^−6^	0.06
Albumin	−5.82 × 10^−5^	1.68	**−9.63 × 10^−5^**	4.14	**−8.64 × 10^−5^**	**1.53**
Creatinine	**2.69 × 10^−4^**	−6.48	8.15 × 10^−5^	−2.83	3.37 × 10^−5^	−0.57
Serum lipid metabolomes						
Apolipoprotein A	−2.39 × 10^−5^	0.70	8.68 × 10^−6^	−0.36	−6.22 × 10^−5^	1.10
Apolipoprotein B	3.82 × 10^−5^	−0.90	−5.14 × 10^−6^	0.17	−1.75 × 10^−6^	0.31
High-density lipoprotein	−9.11 × 10^−6^	0.27	2.20 × 10^−6^	0.10	−1.46 × 10^−5^	0.27
Low-density lipoprotein	7.50 × 10^−6^	−0.18	−1.82 × 10^−5^	0.65	−2.59 × 10^−5^	0.44
Cholesterol	1.15 × 10^−5^	−0.27	−1.48 × 10^−5^	0.51	−3.61 × 10^−5^	0.62
Lipoprotein A	−3.89 × 10^−7^	0.01	2.80 × 10^−6^	0.08	−2.32 × 10^−6^	0.04
Triglycerides	−1.48 × 10^−5^	0.44	1.56 × 10^−5^	−0.54	5.86 × 10^−6^	−0.10
Serum hormone metabolomes						
Insulin-like growth factor-1	−5.81 × 10^−5^	1.39	−2.26 × 10^−5^	0.79	−9.51 × 10^−6^	0.16
Estradiol	−5.44 × 10^−6^	0.18	−1.15 × 10^−5^	0.34	−1.62 × 10^−4^	2.92
Testosterone	6.45 × 10^−5^	−1.70	2.67 × 10^−5^	−0.97	1.60 × 10^−5^	−0.23
Sex hormone-binding globulin	5.76 × 10^−6^	−0.16	−3.48 × 10^−6^	0.14	3.51 × 10^−6^	−0.06
Blood pressure parameters						
Systolic pressure	**−1.27 × 10^−4^**	2.82	**−6.53 × 10^−5^**	2.06	−2.06 × 10^−5^	0.36
Diastolic pressure	**−1.54 × 10^−4^**	3.37	−9.12 × 10^−5^	2.84	−9.18 × 10^−6^	0.16

Abbreviations: ACME, average causal mediation effect; HGS, hand grip strength; m%, mediation proportion. Bold: *p* < 0.05.

## Data Availability

The data presented in this study are openly available in UK Biobank at https://biobank.ndph.ox.ac.uk/ukb/browse.cgi (accessed on 12 November 2021), reference number 91486.

## Supplementary Materials

The following supporting information can be downloaded at: https://www.mdpi.com/article/10.3390/foods14010043/s1, Table S1. Incidence rates of sarcopenia and related symptoms. Table S2. Associations of dietary nitrate with sarcopenia-related parameters. Figure S1. Differences in regression results of dietary nitrate from different sources (Abbreviation: ASM, appendicular skeletal muscle mass; CI, confidence interval; HGS, hand grip strength; SMI, skeletal muscle mass index). Figure S2. Differences in regression results of different sex, age, and CDAI groups (Abbreviation: ASM, appendicular skeletal muscle mass; CDAI, composite dietary antioxidant index; CI, confidence interval; HGS, hand grip strength; SMI, skeletal muscle mass index). Table S3. Characteristics of participants after multiple-imputation. Figure S3. Sensitivity analysis results of logistic regression models (Abbreviation: CI, confidence interval; HGS, hand grip strength; OR, odds ratio; Sarc-Presarc, sarcopenia plus pre-sarcopenia; SMI, skeletal muscle mass index). Figure S4. Sensitivity analysis results (linear regression models; Abbreviation: ASM, appendicular skeletal muscle mass; CI, confidence interval; HGS, hand grip strength; SMI, skeletal muscle mass index).
