# Supplementary material for: The Health Effects of Dietary Nitrate on Sarcopenia Development: Prospective Evidence from the UK Biobank
Source: Foods. 2024 Dec 27;14(1):43. doi: 10.3390/foods14010043 (PMC11719679; doi:10.3390/foods14010043)
Supplement: Supplementary file 1 [file foods-14-00043-s001.zip › foods-3362357-supplementary.pdf]

The Health Effects of Dietary Nitrate on Sarcopenia Development: Prospective Evidence from the UK Biobank

Jigen Na [1,2], Yuefeng Tan [1,2], Yanan Zhang [3], Xiaona Na [1,2], Xiaojin Shi [1,2], Celi Yang [1,2], Zhihui Li [1,2], John S. Ji [1,2] and Ai Zhao [1,2,*]

[1] Vanke School of Public Health, Tsinghua University, Beijing 100084, China.

[2] Institute for Health China, Tsinghua University, Beijing 100084, China.

[3] Oxford Institute of Population Ageing, University of Oxford, Oxford OX1 2JD, UK.

* Correspondence: aizhao18@mail.tsinghua.edu.cn; Tel.: +86-138-1113-1994

Table S1. Incidence rates of sarcopenia and related symptoms.

Outcomes [a]	Overall	Dietary nitrate intake levels grouped by quartiles (mg)				
		Q_1 (0.43–77.07)	Q_2 (77.07–144.30)	Q_3 (144.30–247.82)	Q_4 (247.84–2306.38)	*P*
Sarcopenia						0.293
No	28054 (99.4)	7021 (99.5)	7009 (99.3)	7014 (99.4)	7010 (99.3)	
Yes	175 (0.6)	36 (0.5)	48 (0.7)	43 (0.6)	48 (0.7)	
Sarc-Presarc						0.847
No	23783 (87.9)	5944 (88.0)	5978 (87.9)	5902 (87.3)	5959 (88.1)	
Yes	3286 (12.1)	807 (12.0)	820 (12.1)	857 (12.7)	802 (11.9)	
Low HGS						0.034
No	26445 (93.8)	6573 (93.3)	6601 (93.7)	6644 (94.3)	6627 (94.1)	
Yes	1737 (6.2)	470 (6.7)	443 (6.3)	405 (5.7)	419 (5.9)	
Last HGS (kg)	31.50 ± 10.50	32.59 ± 10.72	31.67 ± 10.52	30.88 ± 10.36	30.84 ± 10.29	< 0.001

Δ HGS (kg)	-3.21 ± 6.77	-3.30 ± 6.94	-3.14 ± 6.79	-3.17 ± 6.70	-3.22 ± 6.62	0.552
Low SMI						0.008
No	25380 (93.6)	6387 (94.4)	6385 (93.7)	6269 (92.7)	6339 (93.6)	
Yes	1734 (6.4)	378 (5.6)	426 (6.3)	497 (7.3)	433 (6.4)	
Last SMI (kg/m^2)	7.50 ± 1.57	7.70 ± 1.65	7.53 ± 1.50	7.40 ± 1.64	7.37 ± 1.44	<0.001
Δ SMI (kg/m^2)	-0.29 ± 0.92	-0.28 ± 1.09	-0.27 ± 0.60	-0.30 ± 1.27	-0.29 ± 0.45	0.205
Last ASM (kg)	21.85 ± 6.11	22.60 ± 6.27	21.97 ± 6.00	21.47 ± 6.24	21.37 ± 5.84	< 0.001
Δ ASM (kg)	-0.98 ± 2.51	-0.96 ± 2.93	-0.95 ± 1.69	-1.01 ± 3.46	-1.01 ± 1.33	0.142
Low walking pace						< 0.001
No	26740 (95.3)	6606 (94.2)	6670 (95.0)	6738 (96.1)	6726 (95.9)	
Yes	1317 (4.7)	405 (5.8)	349 (5.0)	273 (3.9)	290 (4.1)	

Abbreviations: ASM, appendicular skeletal muscle mass; HGS, hand grip strength; Sarc-Presarc, sarcopenia plus pre-sarcopenia; SMI, skeletal muscle mass index.

[a] Categorical variables were shown as numbers (percentage) and tested using the trend chi-square test; continuous variables with normal distribution were shown as mean ± standard deviation and tested using linear trend test.

Table S2. Associations of dietary nitrate with sarcopenia-related parameters.

Nitrate levels	Last HGS (kg)	Δ HGS (kg)	Last SMI (kg/m^2)	Δ SMI (kg/m^2)	Last ASM (kg)	Δ ASM (kg)
	β (95% CI)					
Q_1	0.00 (Reference)	0.00 (Reference)	0.00 (Reference)	0.00 (Reference)	0.00 (Reference)	0.00 (Reference)
Model A [a]						
Q_2	0.15 (-0.08, 0.39)	0.07 (-0.15, 0.30)	0.01 (-0.02, 0.03)	0.01 (-0.02, 0.04)	**0.11 (0.02, 0.20)**	0.03 (-0.06, 0.11)
Q_3	0.07 (-0.16, 0.31)	-0.03 (-0.26, 0.19)	0.01 (-0.02, 0.03)	-0.01 (-0.04, 0.02)	**0.14 (0.05, 0.24)**	-0.04 (-0.12, 0.05)
Q_4	**0.30 (0.07, 0.54)**	-0.11 (-0.34, 0.11)	0.01 (-0.01, 0.04)	0.00 (-0.04, 0.03)	**0.22 (0.12, 0.31)**	-0.03 (-0.11, 0.06)
P-trend	0.028	0.216	0.352	0.487	< 0.001	0.280
Model B [b]						

Q_2	0.14 (-0.09, 0.37)	0.06 (-0.16, 0.28)	0.01 (-0.02, 0.03)	-0.01 (-0.02, 0.04)	**0.11 (0.01, 0.20)**	0.03 (-0.06, 0.11)
Q_3	0.06 (-0.17, 0.29)	-0.04 (-0.26, 0.18)	0.00 (-0.02, 0.03)	-0.01 (-0.04, 0.02)	**0.14 (0.04, 0.23)**	-0.04 (-0.13, 0.04)
Q_4	**0.31 (0.08, 0.54)**	-0.11 (-0.33, 0.11)	0.01 (-0.01, 0.04)	0.00 (-0.04, 0.03)	**0.22 (0.12, 0.31)**	-0.03 (-0.11, 0.06)
P-trend	0.023	0.235	0.356	0.483	< 0.001	0.277
Model C [c]						
Q_2	0.08 (-0.15, 0.31)	0.06 (-0.16, 0.28)	0.00 (-0.03, 0.02)	0.01 (-0.02, 0.04)	0.04 (-0.06, 0.13)	0.02 (-0.06, 0.11)
Q_3	0.00 (-0.24, 0.23)	-0.03 (-0.26, 0.19)	-0.01 (-0.04, 0.02)	-0.02 (-0.05, 0.01)	0.04 (-0.05, 0.14)	-0.05 (-0.13, 0.04)
Q_4	**0.24 (0.00, 0.48)**	-0.08 (-0.31, 0.14)	-0.01 (-0.04, 0.02)	-0.01 (-0.04, 0.02)	0.07 (-0.02, 0.17)	-0.04 (-0.13, 0.05)
P-trend	0.100	0.333	0.541	0.270	0.138	0.172
Model D [d]						
Q_2	0.05 (-0.18, 0.28)	0.06 (-0.16, 0.28)	0.00 (-0.03, 0.02)	0.01 (-0.02, 0.04)	0.03 (-0.07, 0.12)	0.03 (-0.05, 0.12)
Q_3	-0.05 (-0.29, 0.18)	-0.04 (-0.26, 0.19)	-0.01 (-0.04, 0.02)	-0.01 (-0.04, 0.02)	0.03 (-0.07, 0.12)	-0.03 (-0.12, 0.06)
Q_4	0.16 (-0.09, 0.40)	-0.09 (-0.32, 0.15)	-0.01 (-0.04, 0.02)	0.00 (-0.03, 0.03)	0.05 (-0.05, 0.15)	-0.01 (-0.10, 0.08)

P-trend	0.371	0.347	0.525	0.651	0.313	0.505

Abbreviations: ASM, appendicular skeletal muscle mass; CI, confidence interval; HGS, hand grip strength; SMI, skeletal muscle mass index.

Bold: $P < 0.05$.

[a] Model A adjusted for covariates including sex, age, BMI, educational levels, TDI, smoking status, drinking status, and physical activity levels.

[b] Model B adjusted for CVD status and follow-up duration based on Model A.

[c] Model C adjusted for nutrient intakes based on Model B, including dietary energy, carbohydrates, protein, and fat.

[d] Model D adjusted for CDAI based on Model C.

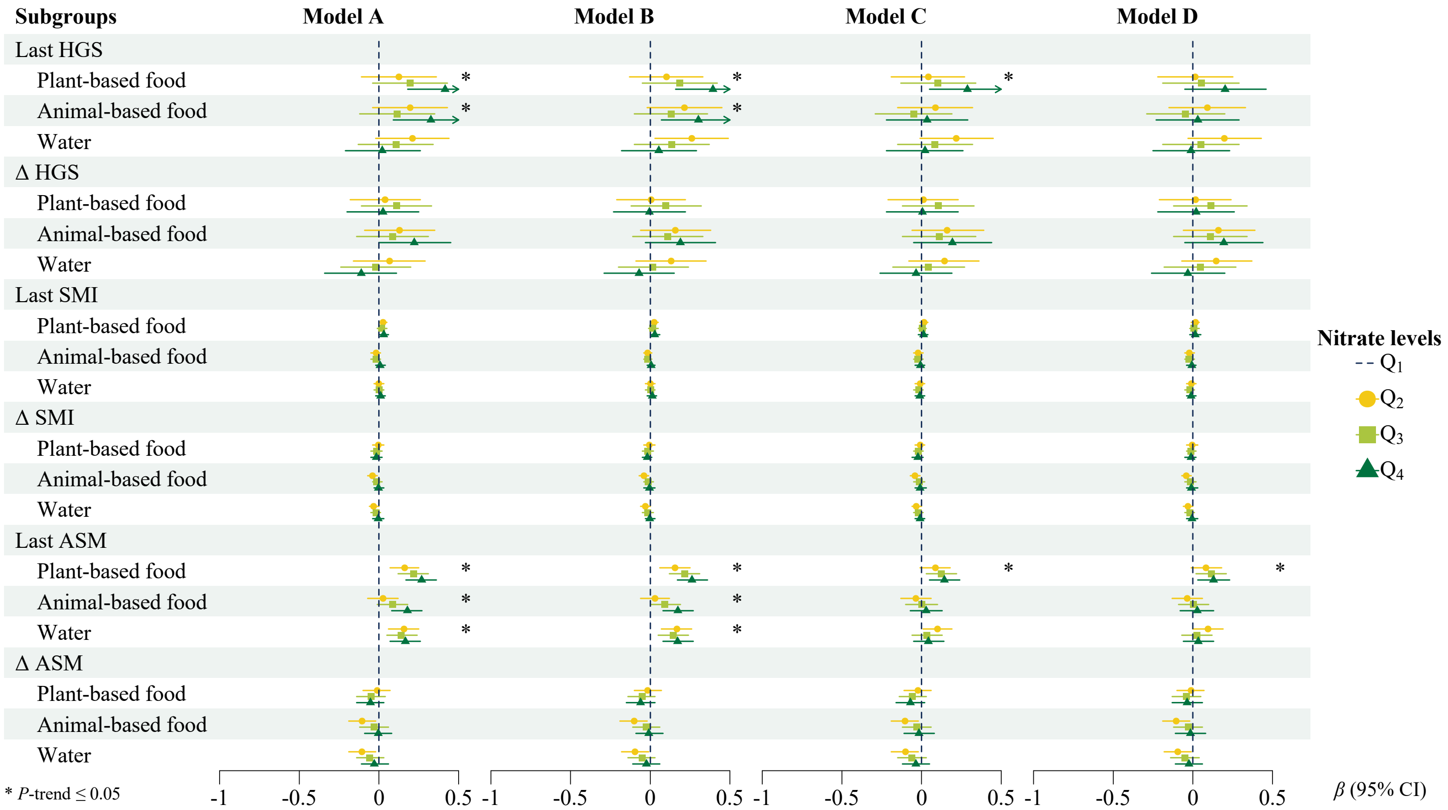

Figure S1. Differences in regression results of dietary nitrate from different sources (Abbreviation: ASM, appendicular skeletal muscle mass; CI, confidence interval; HGS, hand grip strength; SMI, skeletal muscle mass index)

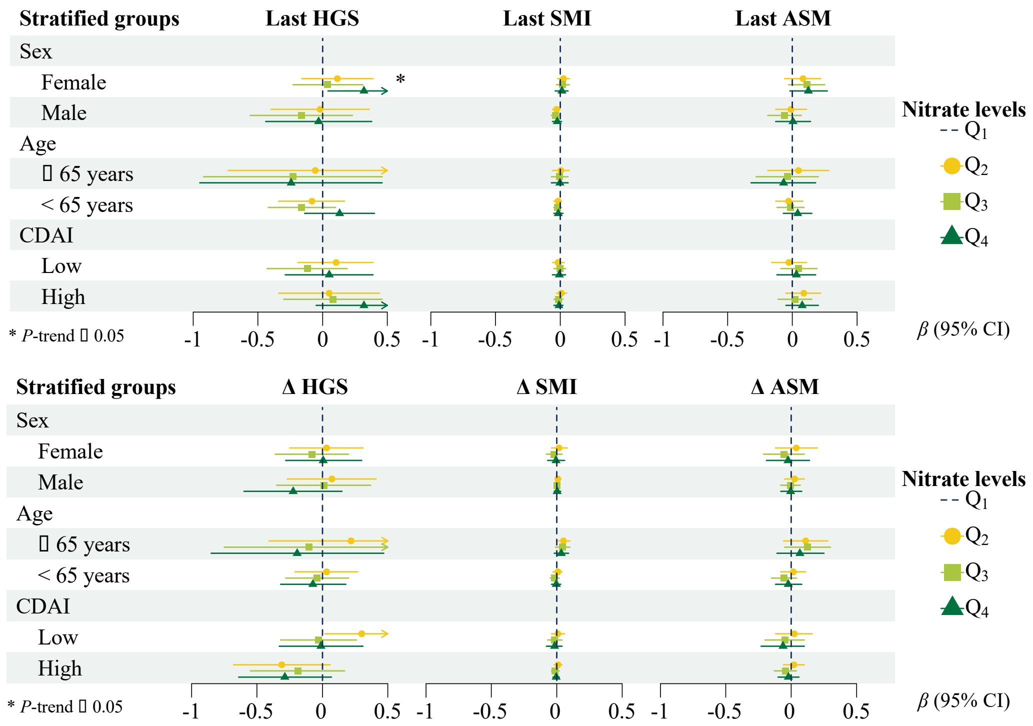

Figure S2. Differences in regression results of different sex, age, and CDAI groups (Abbreviation: ASM, appendicular skeletal muscle mass; CDAI, composite dietary antioxidant index; CI, confidence interval; HGS, hand grip strength; SMI, skeletal muscle mass index)

Table S3. Characteristics of participants after multiple-imputation.

Characteristics [a]	Overall	Dietary nitrate intake levels grouped by quartiles (mg)				
		Q_1 (0.43–77.07)	Q_2 (77.07–144.30)	Q_3 (144.30–247.82)	Q_4 (247.84–2306.38)	*P*
Sex						< 0.001
Female	14601 (51.7)	3141 (44.5)	3551 (50.3)	3896 (55.2)	4013 (56.9)	
Male	13628 (48.3)	3916 (55.5)	3506 (49.7)	3161 (44.8)	3045 (43.1)	
Age (year)	55.47 ± 7.53	54.95 ± 7.53	55.67 ± 7.48	55.57 ± 7.48	55.70 ± 7.59	< 0.001
BMI (kg/m^2)	26.47 ± 4.21	26.77 ± 4.21	26.49 ± 4.22	26.34 ± 4.20	26.27 ± 4.20	< 0.001
Educational levels [b]						
College/University	13753 (48.7)	3043 (43.1)	3414 (48.4)	3532 (50.0)	3764 (53.3)	< 0.001
A/AS levels	3843 (13.6)	943 (13.4)	979 (13.9)	961 (13.6)	960 (13.6)	
O levels/GCSEs	5387 (19.1)	1497 (21.2)	1342 (19.0)	1329 (18.8)	1219 (17.3)	
CSEs	1010 (3.6)	342 (4.8)	250 (3.5)	226 (3.2)	192 (2.7)	

NVQ/HND/HNC	1477 (5.2)	464 (6.6)	374 (5.3)	354 (5.0)	285 (4.0)	
Other qualifications	1285 (4.6)	298 (4.2)	310 (4.4)	324 (4.6)	353 (5.0)	
None of the above	1474 (5.2)	470 (6.7)	388 (5.5)	331 (4.7)	285 (4.0)	
TDI	-2.04 ± 2.62	-2.00 ± 2.64	-2.11 ± 2.57	-2.06 ± 2.62	-1.99 ± 2.64	0.726
Smoking status						< 0.001
Never	17290 (61.2)	4245 (60.2)	4318 (61.2)	4357 (61.7)	4370 (61.9)	
Previous	9406 (33.3)	2317 (32.8)	2379 (33.7)	2340 (33.2)	2370 (33.6)	
Current	1533 (5.4)	495 (7.0)	360 (5.1)	360 (5.1)	318 (4.5)	
Drinking status						0.064
Never	563 (2.0)	124 (1.8)	138 (2.0)	136 (1.9)	165 (2.3)	
Previous	587 (2.1)	159 (2.3)	135 (1.9)	146 (2.1)	147 (2.1)	
Current	27079 (95.9)	6774 (96.0)	6784 (96.1)	6775 (96.0)	6746 (95.6)	
Physical activity						< 0.001

Low level	5000 (17.7)	1465 (20.8)	1276 (18.1)	1174 (16.6)	1085 (15.4)	
Moderate level	12138 (43.0)	3032 (43.0)	3111 (44.1)	3030 (42.9)	2965 (42.0)	
High level	11091 (39.3)	2560 (36.3)	2670 (37.8)	2853 (40.4)	3008 (42.6)	
CVD status						0.051
No	22101 (78.3)	5468 (77.5)	5496 (77.9)	5603 (79.4)	5534 (78.4)	
Yes	6128 (21.7)	1589 (22.5)	1561 (22.1)	1454 (20.6)	1524 (21.6)	
Follow-up time (year)	9.37 ± 2.17	9.37 ± 2.18	9.35 ± 2.15	9.36 ± 2.18	9.39 ± 2.18	0.628
Nutrients intake						
Energy (kcal)	2029.34 [1719.82, 2381.67]	1932.74 [1609.76, 2285.42]	2022.80 [1724.83, 2369.85]	2044.52 [1752.37, 2389.89]	2111.31 [1797.29, 2478.95]	< 0.001
Carbohydrate (g)	249.72 [207.38, 296.07]	235.07 [192.11, 279.86]	244.83 [205.95, 289.45]	251.88 [210.68, 296.56]	267.56 [223.41, 314.67]	< 0.001
Protein (g)	79.42	75.54	79.96	80.50	81.53	< 0.001

	[66.83, 93.20]	[62.31, 89.65]	[67.96, 93.05]	[68.23, 93.70]	[68.53, 95.97]	
Fat (g)	70.47 [55.22, 88.08]	66.20 [50.91, 84.28]	71.13 [55.93, 88.55]	71.68 [57.01, 88.30]	72.76 [57.28, 91.01]	< 0.001
CDAI	-0.30 [-2.51, 2.15]	-2.03 [-4.13, 0.24]	-0.48 [-2.46, 1.65]	0.14 [-1.90, 2.44]	1.17 [-1.10, 3.78]	< 0.001

Abbreviations: BMI, body mass index; CDAI, composite dietary antioxidant index; CVD, cardiac vascular disease; TDI, Townsend deprivation index.

[a] Categorical variables were shown as numbers (percentage) and tested using the trend chi-square test for the ordered variables or the chi-square test for the unordered variables; continuous variables with normal distribution were shown as mean ± standard deviation and tested using the linear trend test; continuous variables with skew distribution were shown as median [P_{25}, P_{75}] and tested using Spearman rank correlation.

[b] Each item involved equivalent levels as described. “Other qualifications” meant other professional qualifications such as nursing and teaching.

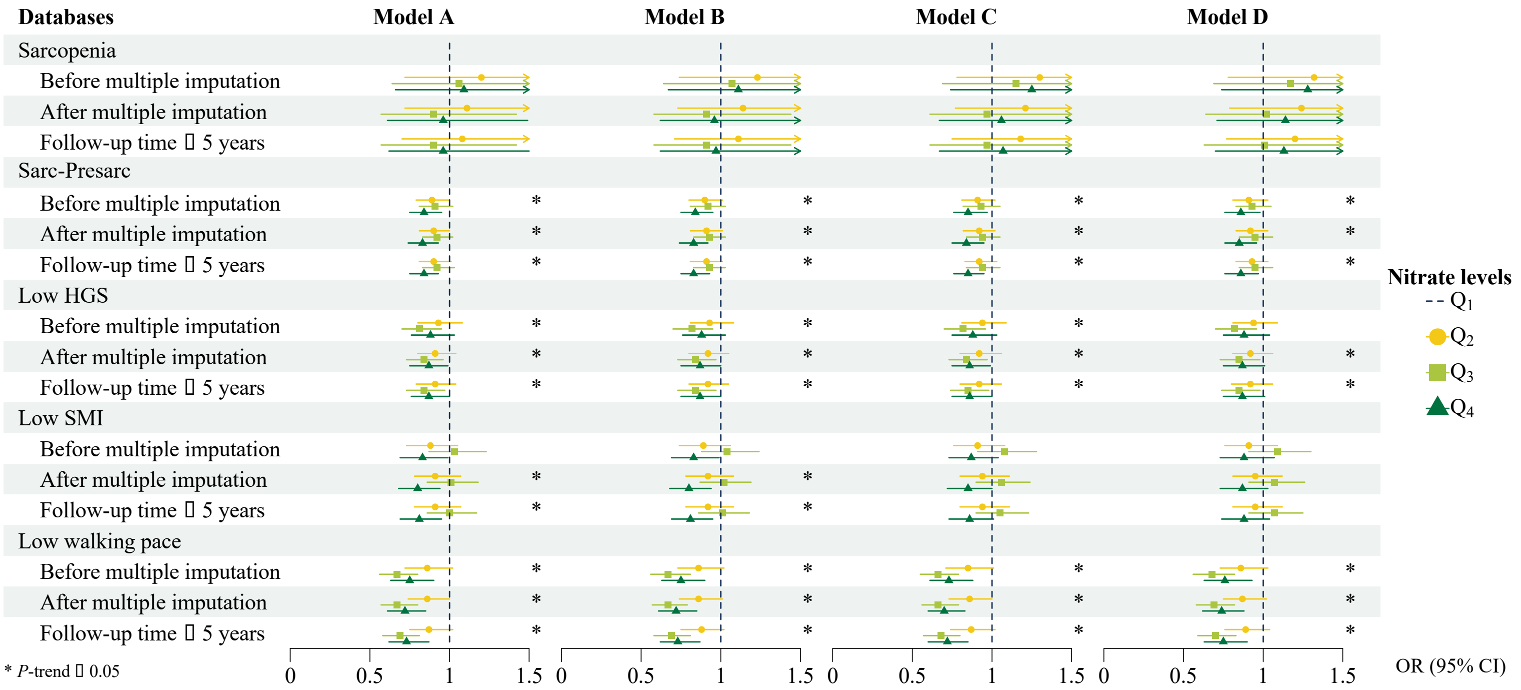

Figure S3. Sensitivity analysis results of logistic regression models (Abbreviation: CI, confidence interval; HGS, hand grip strength; OR, odds ratio; Sarc-Presarc, sarcopenia plus pre-sarcopenia; SMI, skeletal muscle mass index)

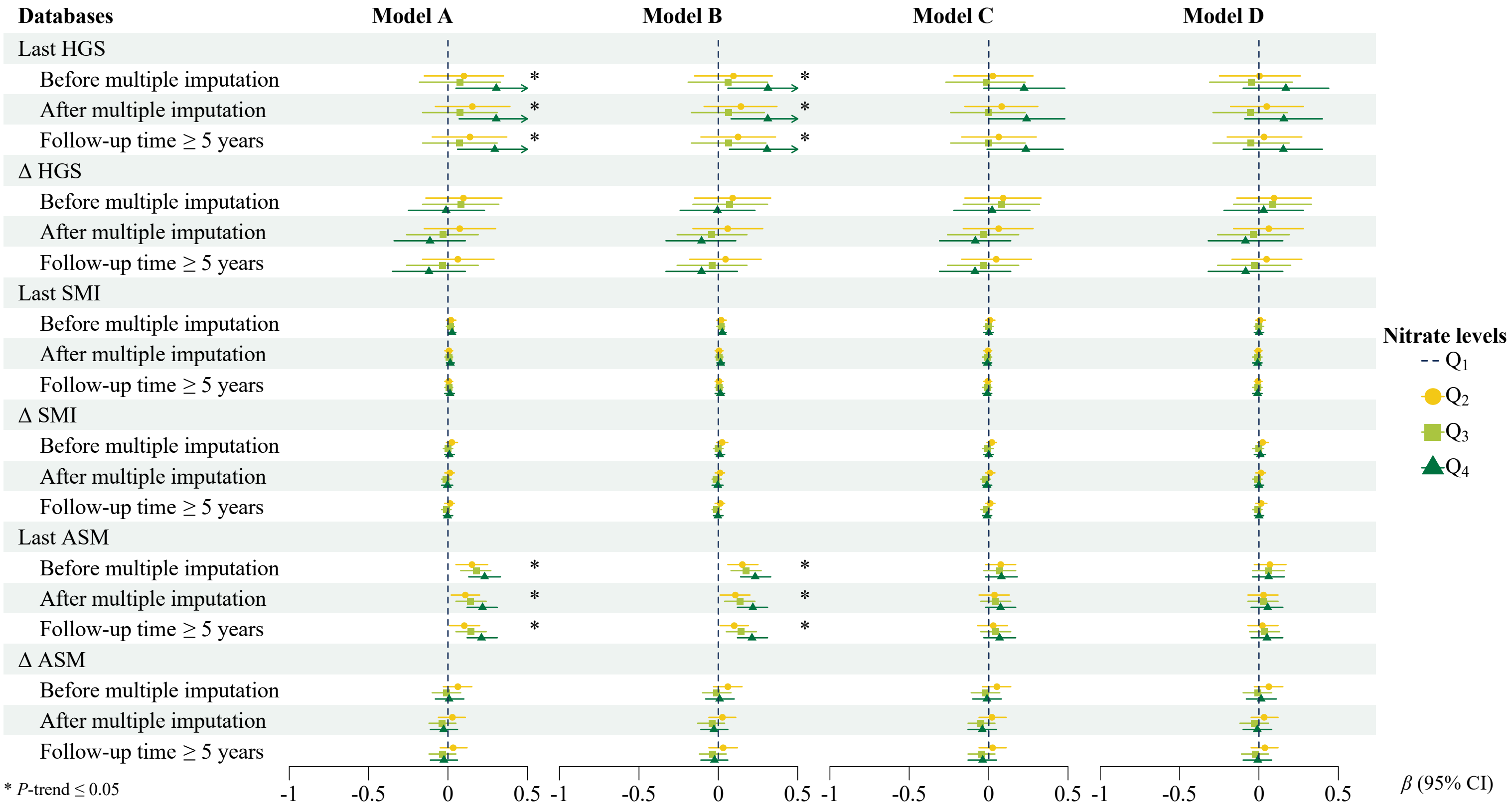

Figure S4. Sensitivity analysis results (linear regression models; Abbreviation: ASM, appendicular skeletal muscle mass; CI, confidence interval; HGS, hand grip strength; SMI, skeletal muscle mass index)
